# Gas formation and biological effects of biodegradable magnesium in a preclinical and clinical observation

**DOI:** 10.1080/14686996.2018.1451717

**Published:** 2018-04-09

**Authors:** Yu-Kyoung Kim, Kwang-Bok Lee, Seo-Young Kim, Ken Bode, Yong-Seok Jang, Tae-Young Kwon, Moo Heon Jeon, Min-Ho Lee

**Affiliations:** a Department of Dental Biomaterials and Institute of Biodegradable Materials, Institute of Oral Bioscience and School of Dentistry (Plus BK21 Program), Chonbuk National University, Jeon Ju, South Korea; b Department of Orthopedic Surgery, Research Institute of Clinical Medicine of Chonbuk National University-Biomedical Research Institute of Chonbuk National University Hospital, Chonbuk National University Medical School, Jeon Ju, South Korea

**Keywords:** Gas formation, magnesium alloys, biodegradable metals, clinical studies, 30 Bio-inspired and biomedical materials, 106 Metallic materials, 211 Scaffold / Tissue engineering / Drug delivery

## Abstract

Magnesium alloys are biodegradable metals receiving increasing attention, but the clinical applications of these materials are delayed by concerns over the rapid corrosion rate and gas formation. Unlike corrosion, which weakens mechanical properties, the gas formation issue has received little attention. Therefore, we evaluated the gas formation and biological effects for Mg implants through preclinical (immersed in Earle’s balanced salt solution and *in vivo*) and clinical studies. The immersion test examined the gas volume and composition. The *in vivo* study also examined gas volume and histological analysis. The clinical study examined the gas volume and safety after Mg screw metatarsal fixation. Gas was mainly composed of H_2_, CO and CO_2_. Maximum volumes of gas formed after 5 days for *in vivo* and 7 days in clinical study. Within the clinical examination, two superficial wound complications healed with local wound care. Osteolytic lesions in the surrounding metaphysis of the Mg screw insertion developed in all cases and union occurred at 3 months. Mg implants released gas with variable volumes and composition (H_2_, CO, and CO_2_), with no long-term toxic effects on the surrounding tissue. The implants enabled bone healing, although complications of wound breakdown and osteolytic lesions developed.

## Graphical abstract

**Figure:**
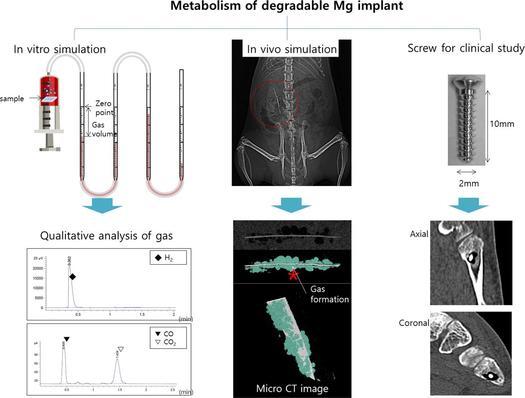

## 1. Introduction

Orthopedic implants are generally made of steel or titanium, and these materials have disadvantages of high stiffness and difficult removal [1]. This issue can be overcome by using degradable implants made of polymer-based materials. However, these materials have poorer mechanical properties than metallic biomaterials since they are associated with foreign objects reaction and osteolysis issues [2]. Magnesium-based materials have been reported as an alternative orthopedic biomaterial. Unfortunately, these implants have a high corrosion rate, which worsens their mechanical properties during biodegradation of material and formation of subcutaneous gas cavities [3]. Thus, recent studies of magnesium-based materials have focused on the enhancement of corrosion resistance and mechanical properties [4–6]. Gas formation around the implant is typically overlooked by researchers since no harmful effects have been reported in preclinical studies [7,8]. Recently, the fast release of hydrogen to form gas cavities was measured in rats using intradermal implantation. The gas composition of a Mg–4Al–0.3Mn alloy was measured *in vivo*, and the main ingredient was found to be N_2_, whereas H_2_ and O_2_ were detected in similar but smaller amounts [9]. The amounts and composition of the gases were similar to the atmospheric composition. The emission of hydrogen gas has been studied by gas collection or by measuring pH changes [10–12]. The analysis of the gas released during biodegradation of magnesium revealed the exclusive presence of H_2_, as predicted by just the chemical theory. These studies did not propose a method for accurately determining the gases by quantitative and qualitative measurement. The formation of gas around the implant was a concern since it can potentially lead to complications such as wound dehiscence, toxic effects on the surrounding tissue and bone defects [13].

The goal of this study was to investigate the formation of gas during both preclinical and clinical magnesium biodegradation. In the preclinical study, the volume and composition of the generated gas were determined from immersing pure Mg into simulated body fluid (SBF) and *in vivo* (via intramuscular implanting of pure Mg in a rat model) [8,14]. In the clinical study, magnesium screws were utilized in metatarsal fracture fixation to evaluate the gas formation and safety of these implants.

## 2. Materials and methods

### 2.1. Materials preparation

The chemical composition of pure Mg ingot (Mg 99.93%, Sincere East Foreign Trade Corp., China) were Mg 99.93, Al 0.0032, Mn 0.0128, Cu 0.0005, Fe0.0017, Si 0.0228 and Ni 0.0003 (wt%). The plate was cut as 15 × 15 × 2 mm, and sequentially ground with SiC abrasive paper from #800 to #2000 for immersion studies. 25 × 5 × 0.25 mm plates were prepared for implantation within the *in vivo* studies.

### 2.2. Immersed in SBF: measurement of gas volume, verification and degradation of Mg

Pure Mg was immersed in an Earle’s balanced salt solution (EBSS) at pH 7.4, and kept at 37 °C under air. Surface analysis and gas formation tests were carried out as illustrated in supplement Figure S1(A), and the volume of gas formation was measured every 24 h.

Mg samples were also placed in low-density polyethylene (LDPE) tedlar bags with EBSS (EBSS-to-Mg volume ratio of 40 ml: 1 cm^3^) and vacuum sealed for 30 days. The gas was collected every 5 days and analyzed with a gas chromatograph (Agilent 7820A, Agilent Technologies, Wilmington, DE, USA) with flame ionization detector (FID) and thermal conductivity (TCD) detectors. The column was pre-heated at 40 °C and operated at 200 °C, and the carrier gas flow rate was maintained. The carrier gas flow rate was controlled (±5%) with an electronic pneumatic control (EPC) module. The detectors were fed with H_2_: 45 ml/min, air: 450 ml/min, N_2_: 25 ml/min (FID) and a reference flow of 10 ml/min (TCD).

To evaluate the concentration of magnesium ion in the EBSS, quantitative analysis of the solution was performed by inductively coupled plasma mass (ICP-MS, Agilent 7500a, Agilent Technologies, Santa Clara, CA, USA). The statistical significance of the results was analyzed by one way ANOVA-test with a *p*-value < 0.05 considered significant.

The surface morphology of the materials was observed by scanning electron microscopy (SEM, JSM-5900, JEOL, Tokyo, Japan) after immersion in EBSS for 30 days. The chemical composition of the by-products formed on the surface of pure Mg after immersion was analyzed by energy dispersive X-ray spectroscopy (EDX, Oxford Instruments, Abingdon, UK).

Potentiodynamic polarization was conducted using Potentiostatic/Galvanostatic 2273 (AMETEK, Oak Ridge, TN, USA) to measure corrosion potential and current density. An Ag/AgCl electrode (Orion, Beverly, MA, USA) and a platinum plate electrode served as reference and counter electrodes, respectively. The potentiodynamic polarization scans were carried out at a sweep rate of 3 mV·min^−1^ in the potential range from −1.8 to −0.9 V. The electrochemical impedance spectroscopy (EIS) tests were conducted with a potential perturbation of 10 mV around the open circuit potential in a frequency range of 100 kHz to 100 mHz. Impedance data were presented as Nyquist plots and fitted with ZSimpWin 3.22d^®^ software.

### 2.3. *In vivo*: surgery, measurement of gas volume and degradation of the Mg implant

All experimental protocols were approved by the Institutional Animal Care (CBNU 2015-034) and the Committee of Chonbuk National University following the guidelines of the National Institutes of Health (USA). Twenty-four 8-week-old male Sprague-Dawley rats (weight: 250–270 g) were divided into two groups: the implantation (*n* = 18) and the sham surgery control (*n* = 6). The animals were anesthetized and the surgical site was shaved and prepped by alternating betadine and 70% alcohol scrubs; all animals received a subcutaneous penicillin injection. Using aseptic techniques, a 25-mm posterior longitudinal incision was made 10 mm from midline and a dorsolumbar muscle-splitting approach was used followed by intramuscular Mg implantation, as illustrated in supplement Figure S1(B). The sham surgery control group was sutured without getting the specimen after incising the dorsolumbar site. The fascia was closed with absorbable suture, and the skin was closed with a non-absorbable monofilament suture. Postoperatively, the animals were allowed to eat and drink (ad libitum) while monitoring their health status on a daily basis. Scanning by micro-computed tomography (micro-CT; SKYSCAN 1076, Skyscan, Aartselaar, Belgium) occurred at 5, 15, and 30 days to measure gas volume and determine the degradation of the samples. Four rats (3 implanted, 1 control) were sacrificed every 5 days for tissue analysis (liver, kidney and surrounding tissues). The collected tissue was crushed with 1 ml of distilled water as 0.1 g and maintained for 2 h to extract the Mg ions. Subsequently, it was centrifuged (660 g, 10 min), and the supernatant fluid (250 μl) was collected. The concentration of Mg ions in each tissue was quantitatively analyzed by ICP-MS. The concentrations of Mg ion were calculated using the following formula;

Rateofionconcentration(%)=implantedgroup(ppm)/controlgroup(ppm)×100

Statistical significance was analyzed using one-way ANOVA-test except for the negative group. *p* value <0.05 was considered significant. The collected tissue was also fixed with a 10% formaldehyde solution, paraffin-embedded and then the deparaffinized 5 μm slices were stained with hematoxylin and eosin (HE) and microscopically examined. After taking out the implanted samples, the surface morphology of the Mg plates was observed by SEM, and the chemical composition of the by-products was analyzed by EDX.

### 2.4. Clinical observation

The cortex Mg-5Ca screws (HP Mg, more than 99.98 wt.%; 5 wt.% Ca) with an outer diameter of 2.0 mm, inner diameter of 1.6 mm, and length of 10.0 mm (Figure S1(C)) were manufactured by U&I corporation in South Korea. Following approval by Chonbuk National University Hospital Institutional Review Board, the study was performed in accordance with relevant guidelines and regulations. The study of biodegradable Mg alloy screws was conducted retrospectively for patients 20 years or older having metatarsal or midfoot fractures requiring internal fixation. Twenty-two patients voluntarily participated and informed consents were obtained. The exclusion criteria were severe infections, soft tissue injuries, open fractures, major systemic diseases and kidney disease with a blood creatinine level higher than 1.4 mg/dl, or a disease that could affect the bone healing process. Demographic data revealed a mean age of 39 ± 16, with 13 males and 9 females. Follow-up was performed at 1, 4, 8, 12, 24 weeks and 1 year. X-ray and low-dose radiation CT were performed to evaluate the bone healing process and the gas volume. The mean gas volume was measured by a manual tracing technique using the mean region of interest tool available in the Picture Archive and Communication System for volume measurement.

The splint was removed after 1 week and weight bearing was permitted after 1 year. Upon confirmation of the bone union at 3 months, the patients were released to full activity. Along with the bone union assessment at 12 months, the American Orthopaedic Foot and Ankle Society (AOFAS) Ankle-Hindfoot Score, and the visual analog scale (VAS) were also measured. The AOFAS score was calculated using a 30-item questionnaire answered by the patients before and after surgery. The pain VAS was completed using 0–10 scores. The statistical significance was analyzed by paired *t*-tests, and a *p*-value < 0.001 was considered significant.

## 3. Results

**Figure 1.**
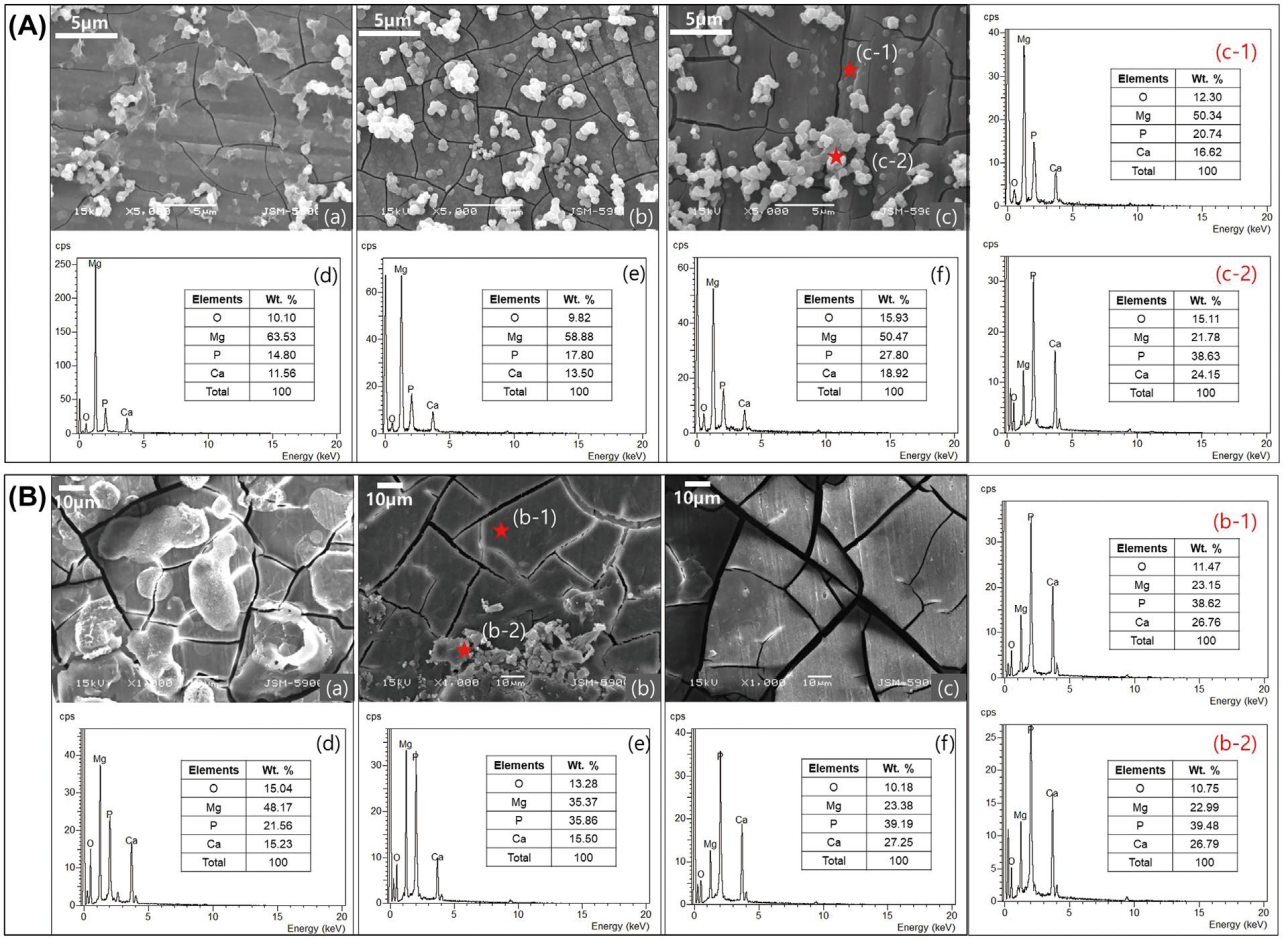
(A) SEM images of the Mg surface after immersion in EBSS for (a) 5, (b) 15, and (c) 30 days, EDX spectra after (d) 5, (e) 15, (f) 30 days, and EDX spectra of points (c-1) and (c-2) from image (c); (B) SEM images of the Mg surface after implantation to rat for (a) 5, (b) 15 and (c) 30 days, EDX spectra after (d) 5, (e) 15, (f) 30 days and EDX spectra of points (b-1) and (b-2) from image (b).

**Figure 2.**
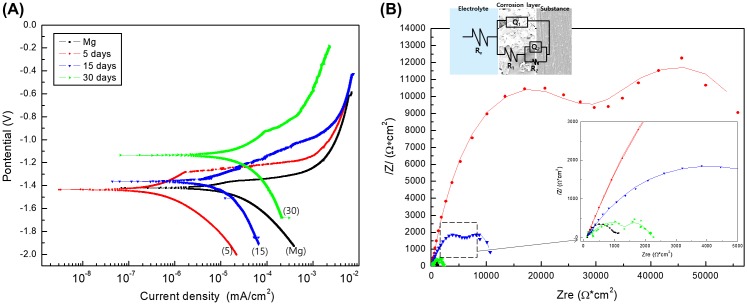
(A) Potentiodynamic polarization curves and (B) Nyquist plots of electrochemical impedance spectroscopy data after immersion for 5, 15 and 30 days in EBSS.

The surface of the Mg implant after post-immersion for 5 days showed circular particles on the oxide film with microcracks in Figure 1A(a). After 15 days of immersion (Figure 1A(b)), the thickness of the oxide film increased and the composite structures contained globular and planar apatite layers. The particles formed irregular patches as a result of continuous corrosion after 30 days (Figure 1A(c)). The intensity of the Mg peak decreased with the immersion time, while the peaks corresponding to P, Ca, and O increased in intensity, as shown in Figure 1A(d)–(f). After 30 days, the composition of magnesium surface as Figure 1A(c-1) was similar to the composition ratio of the entire surface (Figure 1A(a) and (b)), but the circular particles adhering to the surface increased in size according to time, and consisted of P and Ca components as Figure 1A(c-2).

Figure 2 shows the DC polarization and electrochemical impedance spectroscopy (EIS) results of pure Mg for different EBSS immersion periods. The corrosion current density (*I*_corr_) acquired from the cross point of the Tafel slopes by the anodic and cathodic (*β*_a_ and *β*_b_, respectively) of the DC polarization curves are summarized in Table 1. As shown in Figure 2(A), the corrosion potential (*E*_corr_) gradually increased as the immersion time in EBSS increased, and the current density decreased but rebounded again after immersion for 5 days. Thus, the corrosion resistance increased only for the initial 5 days, and ultimately the corrosion resistance after 30 days was found to be lower than that of the non-immersed Mg. Figure 2(B) shows changes in the Nyquist plot from EIS according to the immersion time. Variations in the corrosion resistance according to the immersion time were determined from the Nyquist plots and fitted to equivalent circuit models (ZSimpWin software). The quality of fitting was judged by the chi-square value in Table 1. In the equivalent circuits, the electrolyte resistance values (*R*_*e*_) were not significantly different from each other, whereas the coating resistance (*R*_1_) caused by the corrosion products tended to increase up to 5 days of immersion and then decrease. The charge transfer resistance (*R*_2_) caused by the charge transfer process also tended to decrease as *R*_1_ after 5 days, but had a higher value than that of the non-immersed Mg due to the formation of an oxide film. The immersion polarization resistance (*R*_*p*_) acquired by a summation of *R*_1_ and *R*_2_ increased to its highest value after 5 days, but decreased again with further immersion time. Therefore, the trend of corrosion resistance shown by these results was consistent with the corrosion rate observed in the DC polarization curve (Figure 2(A)).

**Table 1.** Summary of results fitted from potentiodynamic polarization curves and Nyquist plots after immersion periods for 30 days.

Time (days)	*E*_corr_ (V)	*I*_corr_ (μA/cm^2^)	*R*_e_ (Ωcm^2^)	*Q*_1_ ×10^5^ (Ω^−1^ cm^−2^s^n^)	*n*_1_	*R*_1_ (Ωm^2^)	*Q*_2_ ×10^5^ (Ω^−1^ cm^−2^s^n^)	*n*_2_	*R*_2_ (Ωcm^2^)	Chi-square
0	−1.42	7.899	120.6	0.059	0.8733	831.8	324.8	0.678	289.6	0.00414
5	−1.44	0.3325	104.8	0.088	0.7155	31030	2.161	0.65	35400	0.00804
15	−1.36	4.361	112.4	0.621	0.5811	7360	9.859	0.815	11252	0.00292
30	−1.14	11.82	139.6	0.002	0.6512	1517.5	1.653	0.509	2293	0.00796

**Figure 3.**
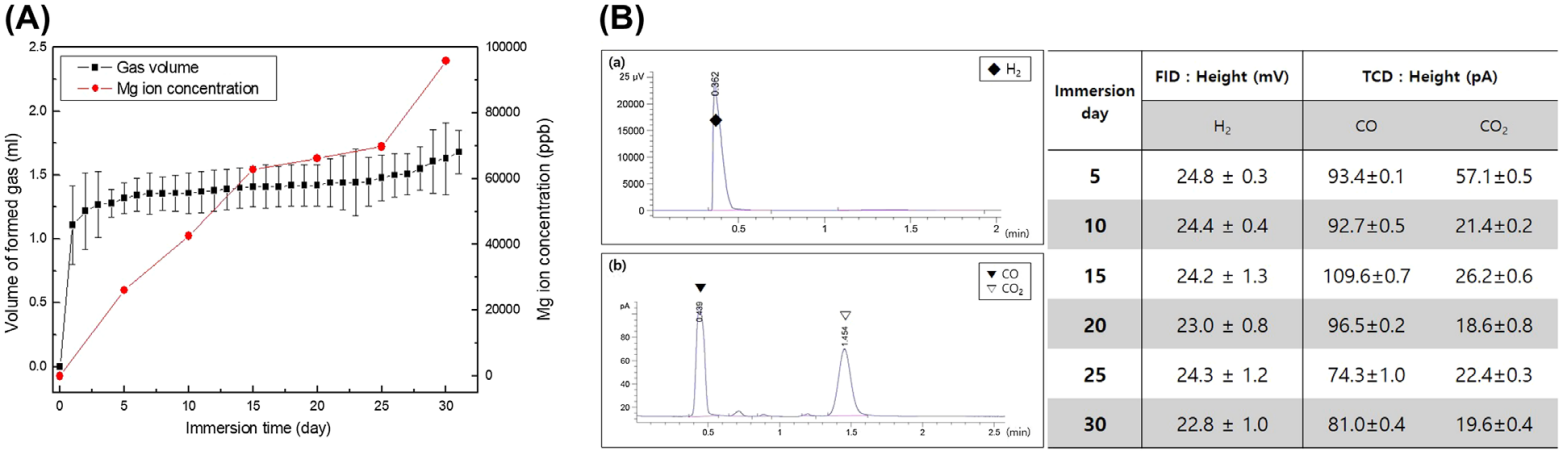
(A) The amount of formed gas measured daily for 30 days and Mg ion concentration measured every 5 days during immersion in EBSS for 30 days, and (B) Gas component analysis measured by (a) FID and (b) TCD during immersion in EBSS for 30 days.

The volume of gas increased quickly during the first 5 days, and moderately increased thereafter because of the formation of corrosion products and surface oxide films as shown in Figure 3(A). The concentration of Mg ions increased as a result of the local corrosion after 25 days, and the gas formation started to rebound again. The amount of Mg ions uniformly increased up to 15 days and then stabilized as the result of the gas formation. But the Mg ion concentration increased fast after 25 days, because the corrosion layer formed on the surface was destroyed, and the corrosion progresses rapidly as the magnesium was locally exposed. Similar to the results of the potentiodynamic polarization test in Figure 2(A), the growth and dissolution of the oxide layer formed on the magnesium surface repeatedly changed the ion concentration of magnesium and the gas release rate.

The gas composition detected by the reaction of pure Mg in the EBSS was analyzed by gas chromatography (GC) as shown in Figure 3(B). The main gas formed in all implant groups had a major hydrogen peak that showed an even height by the FID irrespective of immersion time. Analysis of traces of residual gas through the TCD detected CO and CO_2_. The amount of CO was somewhat higher than that of CO_2_. There were no significant changes in the detected amount of CO with increased immersion time, whereas the amount of CO_2_ rapidly decreased after immersion for 10 days and then remained constant up to 30 days (Figure 3B(b)).

The component ratio of magnesium surface after *in vivo* test was similar to that of *in vitro*. The Mg surface after *in vivo* in Figure 1(B), the ratio between P and Ca remained nearly constant. The only observed change is that the *in vivo* result in Figure 1B(a)–(c) is a decrease in Mg concentration and an increase in oxygen compared to the oxide film formation in EBSS. On the other hand, the *in vivo* test showed that the presence of P and Ca on the stable oxide film could be advantageous since the amount of body fluid in contact with the Mg surface and the corrosion rates were lower as compared to the SBF environment (Figure 1B(d)–(f)). Magnesium obtained under the rat skin was not observed to be formed apatite particle as *in vitro*, and the partially adhered particles Figure 1B(b-2) showed the same composition ratio as the cracked oxidized surface (b-1).

**Figure 4.**
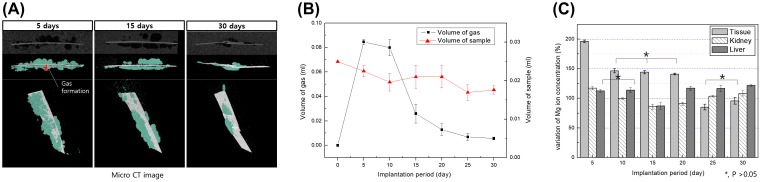
(A) Micro CT images of Mg plate (white color) and gas pocket (light blue) after implantation in rats for 5, 15 and 30 days, (B) Volume of the Mg plate and gas pocket after implantation in rats for 30 days, and (C) Mg ion concentration in muscle tissue attached to the specimen, kidney and liver measured by ICP-MS.

Gas rapidly formed around the implant during the first 5 days (volume = 0.084 ml) as asterisk (*) mark in Figure 4(A). The volume then decreased by 28% after 10 days and to 0.005 ml after 30 days (Figure 4(B)). The gas formation is different from *in vitro* results. Gas formation was greatly increased by the initial abrupt corrosion, but gradually decreased after 10 days. The formed gas is absorbed into the body, the corrosion rate is delayed due to the corrosion product on the magnesium surface, and the gas formation rate is also decreased, so that the gas in the body is gradually decreased. The amount of magnesium volume decreased with the corrosion initial time, although the sample volume seemed to increase temporarily due to the formation of corrosion products, and as a result, it gradually decreases as it is absorbed in the body.

The detection of Mg ions within the liver, kidney and surrounding soft tissue was carried out at different implanted times by ICP-MS as shown in Figure 4(C). The concentration of Mg ions in the tissues surrounding the implantation increased by 196% during the first 5 days after implantation, and by 140–146% after 5–20 days. After 25 days the concentration of Mg ions decreased to initial values and did not significantly change. The concentration of Mg ions in the kidney decreased up to 15 days and increased thereafter. In the liver, the concentration of Mg ions was within the range of the control group during the first 10 days after implantation (*p* > 0.05) and increased slowly up to 30 days.

**Figure 5.**
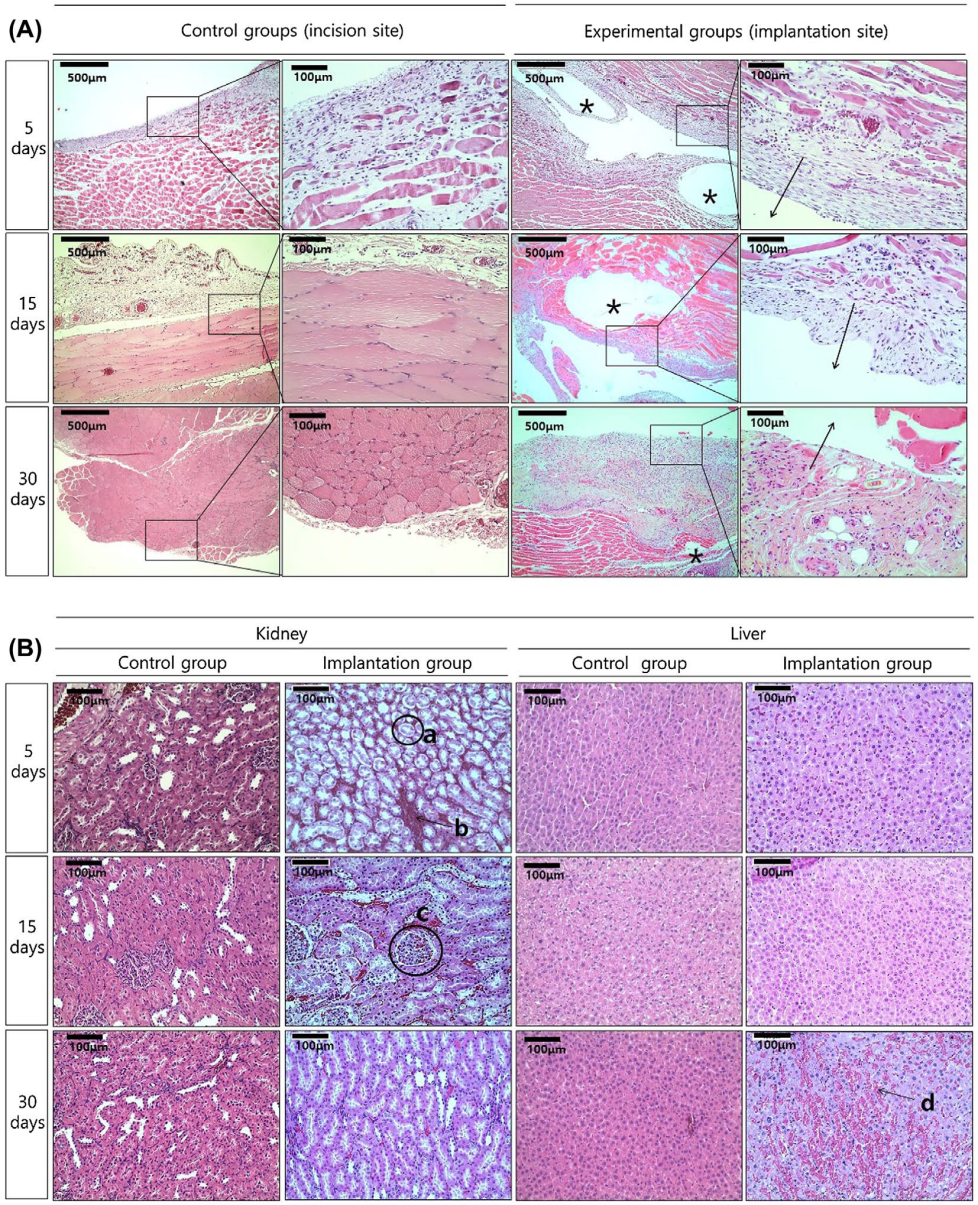
HE stained histological images of (A) the tissue attached to the specimen (The direction of the arrows is the implantation site, and gas pockets mark as asterisk, *), and (B) the kidney and liver tissues after implantation.

Figure 5(A) shows histologic images of tissue around the implant stained with hematoxylin and eosin. The specimens of all groups showed histologic reactions caused by Mg implantation from the skin to the subcutaneous layer sectioned vertically from the skin. The direction of the arrows at the implanted site in the high-magnification image indicates the direction of the implanted Mg, and the opposite direction is towards the inner tissues. For the control group, muscles in the left lumber region of rats were sectioned using the same conditions as the implanted group. After 5 days of implantation, nucleus formation was concentrated around the wounds of both the control and the implanted groups. The muscle fibers were divided and decreased in size due to tissue proliferation between the muscle fibers. In the implanted group, hydrogen gases formed as the Mg dissolved, creating small and multiple bubble pockets (marked with an asterisk, *). During the initial 5 days, fibroblasts densely concentrated around the bubble pocket, and then formed around the incision. After 15 days, all groups still had dense nuclei around the wounds, but the muscle fibers in the incision regions showed reduced separation and a shape similar to that of normal tissues. Although bubbles were present continuously in the implanted group, the dense nuclei that gathered around the wounds as films disappeared and changed into normal muscle fiber tissue. After 30 days, the muscle fiber tissue in the incision regions showed increasing density and nuclei concentration was found. Although the number of gas bubbles and their volumes were reduced considerably in the implanted group, divided muscle fiber tissue was observed due to continuous tissue proliferation in the layer of muscle fibers.

Histologic analysis of the kidney and liver tissue after Mg implantation is shown in Figure 5(B). The kidney consisted of mainly proximal tubules (a), and mitotic epithelial cells were formed around the tubules as a brush border with blood cells (b) filling the area between the tubules. The glomerulus (c) showed a globular capillary network of 80- to 110-μm diameters surrounded by the Bowman capsule, which had the same morphology in the control and the implanted groups. The kidneys in both groups were visually similar regardless of treatment time, and their tissue shapes were not significantly different between groups. The liver tissue in the control group showed typical liver cell shapes during the entire test period. In the hepatocyte cells, well-developed rough endoplasmic reticulum, mitochondria, and glycogen granules were observed. In the implanted group at 5 days, histologic changes in the liver tissue were observed, with a small area of vacuolization shown in Figure 5B(d). No inflammation or deformation was observed in the 30-day immersion group.

**Figure 6.**
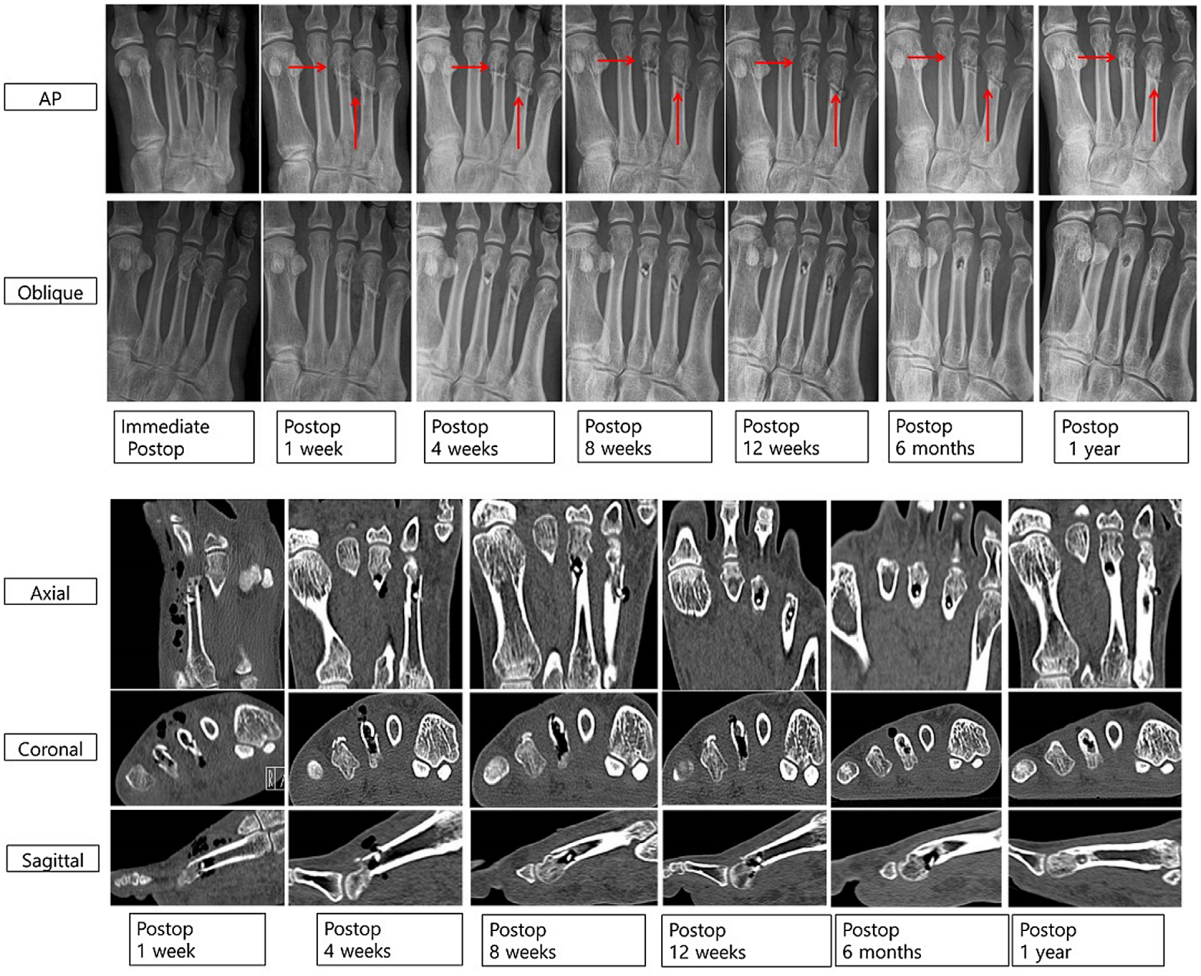
Foot AP and oblique X-ray images of gas formation by magnesium screw (red arrows), and axial, coronal, and sagittal CT scan images of air bubbles surrounding magnesium screw inserted into metatarsal fracture for 12 months.

All patients had bony union at 3 months and after 6 months, the metatarsal fracture was completely healed with a small radiolucent area in the screw insertion site (Figure 6). Foot anteroposterior (AP) and oblique X-ray images show that the radiolucent lesion on 3rd and 4th metatarsal neck fractures means gas formation by magnesium screw (red arrows), which decreases over time. Cortical continuity was observed and the diameter of the inserted Mg screw was significantly reduced. Axial, coronal, and sagittal CT scans show multiple air bubbles surrounding magnesium screw inserted into metatarsal fracture, which decrease over time. Especially, postoperative 12 weeks and 6 months CT scans show small amounts of gas in soft tissue as compared to postoperative 1, 4, and 8 weeks. Although the amount of gas in bone decreases over time, it remains regardless of the complete healing on cortex of metatarsal fracture.

The mean gas volume in both soft tissue and bone (supplement Figure S2 and Table S2) reached a maximum after the first week and decreased thereafter (paired *t*-test, *p* < 0.001). The gas volume in the soft tissue was higher than that in the bone. There was no significant differences in gas volume by age or gender (*p* = 0.756, 0.467). Complications of wound dehiscence occurred in 2 cases (i.e. superficial skin necrosis along the incision margin), and these resolved with local wound care. VAS decreased over time (preoperative: 7.1 ± 0.8 vs. 1 year postoperative: 1.8 ± 0.8, *p* < 0.0001). The AOFAS score (Table S3) increased over time (preoperative: 41 ± 8 to 1 year postoperative 89 ± 4, *p* < 0.0001).

## 4. Discussion

The current Mg studies are focused on retarding the corrosion rate and enhancing the mechanical strength, with little interest in the gas formation during degradation. Most researchers reported gas formation during degradation without specific complications in the preclinical studies [10–12,14,15]. It is necessary to analyze the formed gas more accurately to understand the biodegradation mechanism and its effect on the histologic reaction. In this study, EBSS was used for the immersion test to investigate the amount and composition of gas formed during degradation of Mg in the body, it specially showed the most similar bio reaction and stable corrosion progress to *in vivo* results among various simulated body fluids tested in a previous study [16].

Within our study, gas formation quickly developed during the first day and then decreased thereafter. A stable oxide film comprising of P and Ca was formed on the surface of Mg after 5 days of immersion, which resulted in a significant reduction of the gas evolution rate. The amount of dissolved Mg ions steadily increased up to 15 days. In the surface of Mg during immersion test, the particles with a length of 1 to 2 μm became more numerous and grew in size [17]. It is a kind of apatite, a Ca-P compound, which grows over time and covers the entire surface with oxides [18,19]. In many past studies, magnesium in SBF has formed not only Mg oxide but also Ca and P, which deposited on the coating layer as various amorphous apatite structures including hydroxyapatite (Ca_10_(PO_4_)_6_(OH)_2_), brushite (CaHPO_4_·2H_2_O) and octacalcium phosphate (Ca_4_(HPO_4_) (PO_4_)_2_·25H_2_O) [20–22].

The different gas formation rates and dissolution rates of Mg ions can be explained as follows:[23]

(1) $$\text{Mg}\;+2{\text{H}}_{2}\text{O}\;\to \;\text{Mg}{\left(\text{OH}\right)}_{2}+{\text{H}}_{2}$$

Gas formation initially occurs when Mg and H_2_O in the body fluid come into contact [24]. The rate of H_2_ generation increased rapidly for 5 days and remained constant as shown in Figure 3(A). The other product of the reaction is Mg(OH)_2_ which precipitates, and MgCl_2_ is also formed on the surface due to the presence of chloride ions in the simulated body fluid [25]. In other words, the formed Mg(OH)_2_ is readily converted to the more soluble MgCl_2_ by the chemical reaction of Mg(OH)_2_ + Cl^− ^→ MgCl_2_, leading to magnesium corrosion.

The amount of dissolved Mg ions did not increase as fast as gas formation at initial stages, but constant dissolution occurred. Continuous Mg degradation can occur faster during EBSS immersion as compared to *in vivo* because the convection and diffusion are higher in EBSS. The gas formed around the magnesium implant is mainly absorbed and reacted with body fluids and moves into the capillary and then it escape through the lung [26]. The gas bubbles formed in the simulated fluid obtain a pressure similar to atmospheric pressure [11,27]. For *in vivo*, the pressure on the formed gas will be different for each tissue site.

As shown in the SEM image of Figure 1(B), the protective magnesium oxide/hydroxide layers (MgO or Mg(OH_2_)) are distributed throughout the magnesium surface as a spontaneous passivation, but they have a brittle ceramic structure. The process of exposing and corroding magnesium is repeated. Cracks can be generated at chemical bonding or drying stages depending on various aspects such as MgO (reactivity), interaction with other compounds (e.g. Ca, P, Na) [28]. The Ca/P ratio significantly increased within a restricted diffusion environment during *in vivo* tests. The gas pockets which resulted in a separation of the tissue layers and bloodstream blockade [29], slowed corrosion with a more stable calcium phosphate layers as compared to *in vitro* conditions. The results of potentiodynamic polarization and EIS after immersion in EBSS showed that the Mg immersed for 5 days had the highest corrosion resistance due to the formation of a dense and stable corrosion product on the surface, but local corrosion was accelerated as the immersion time increased. This is because many cracks in the corrosion products occurred as the thickness of the corrosion product increased, which resulted from a large Pilling–Bedworth ratio (MgO/Mg is 0.81, while Mg(OH)_2_ is 1.77) caused by a density difference between the oxide and the pure Mg base metal. In addition, the corrosion current density increased again because the corrosion product was partially separated from the surface. Although the group immersed for 30 days showed low corrosion resistance, the corrosion potential in the potentiodynamic polarization curve was the highest. Because the electrode potential of the corrosion product generated in the simulated body fluid was higher than that of pure Mg, the corrosion potential increased according to the growth of corrosion product on the surface. This means that the protective film (i.e. magnesium hydroxide or magnesium oxide) that was initially deposited on the surface was subsequently broken down by chloride ions [30].

When Mg is immersed in EBSS for a short period of time, in addition to the corrosion, a multilayer structure consisting of corrosion products such as Mg(OH)_2_, MgCl_2_, and apatite is generated. Such a multilayer has two capacitive loops in the Nyquist plot [31]. When the oxide film is assumed a two-layer film which are one inner barrier layer and an outer porous layer, the equivalent circuit for this case is shown in Figure 2(B). In the first curve (low X axis), *R*_*e*_ is resistance of solution, and *R*_1_ represents the resistance of corrosion products. In the second curve, *R*_2_ represents the resistance caused by the charge transfer process [32]. Total *R*_*p*_ is associated to the outer oxide layer resistance (*R*_1_) and sealing closes (*R*_2_) partially the film promoting a better behavior of corrosion resistance [33]. *R*_1_ is a value resulting from resistance due to the conduction flow of ions passing through the coating layer, and it is connected in parallel with the constant phase element (*Q*_1_) of the coating layer. *R*_2_ is a coating layer/metal system (*Q*_2_) of the double layer at the coating layer/metal interface, and is connected in parallel. When the solution penetrates into the unstable oxide layer, *R*_1_ increases and *Q*_1_ is affected relatively. The lower *R*_1_ is due to micro-defects such as pores and cracks generated inside by the various influences such as Cl^−^ ions in the magnesium corrosion process. Since the non-immersed Mg does not form a definite oxide layer, the impedance values of the capacitive arc caused by the layer capacitor *Q*_1_ and the charge transfer resistance *R*_1_ are not distinguished from each other and mixed. Mg immersed with SBF was formed an oxide layer, which showed a capacitive arc with two semicircles. The immersion polarization resistance (*R*_*p*_) acquired by a summation of *R*_1_ and *R*_2_, and it increased to its highest value after 5 days, but decreased again with further immersion time. This indicates that the formation and destruction of oxides occur iteratively. After 30 days, *I*_*corr*_ was slightly higher than the magnesium sample, but the corrosion potential was high. As the result of EIS, double layer is still formed like the existence of clear *R*_1_ and *R*_2_, so it is considered that the surface oxidation tendency is lowered and the corrosion is reduced [34,35].

FID is a type of GC used to detect ions formed during the combustion process of organic compound in a hydrogen flame, in which the gas to be measured flows into the detector gas. Until now, Mg degradation and gas formation in the body have been predicted based on chemical theories, such that the generated gas was specified as single H_2_ gas and only changes in its volume were measured using the gas collection method [15]. In the FID measurement, H_2_ was detected as a single peak, and residual micro-gases were measured using TCD. In a previous study that analyzed gases using mass spectrometry while forming a gas cavity in rats [8], only H_2_ and CO_2_ were detected. McBride measured the gas composition generated upon implantation of a Mg–4Al–0.3Mn alloy into an animal body for 40 days and identified the gas composition as 5.6% CO_2_, 6.5% O_2_, 7.3% H_2_, and 80.6% N_2_, which is similar to the atmospheric composition [3]. On the other hand, the present study detected H_2_, CO_2_ and CO, which is different from the above results. According to the previous study [36], Ca^2+^, Mg^2+^ and ${\text{HCO}}_{3}^{-}$ in the body fluid (Table S1) formed H_2_CO_3_ by reaction with H_2_O. As one of the processes of biological metabolism, CO_2_ can be formed via the following cycle:

Carbonic acid will dissociate:

(2) $${\text{HCO}}_{3}^{-}+{\;\text{H}}^{+}\Leftrightarrow {\text{H}}_{2}{\text{CO}}_{3}$$

(3) $${\text{H}}_{2}{\text{CO}}_{3}\Leftrightarrow {\text{CO}}_{2}+{\;\text{H}}_{2}\text{O}$$

Or bicarbonate and free hydrogen ions are formed as follows:

(4) $${\text{HCO}}_{3}^{-}\Leftrightarrow {\text{CO}}_{3}^{2-}+{\;\text{H}}^{+}$$

Considering the relationship between H_2_CO_3_, ${\text{HCO}}_{3}^{-}$ and ${\text{CO}}_{3}^{2-}$ in the aqueous carbonate system, it is stabilized with ${\text{HCO}}_{3}^{-}$ → ${\text{CO}}_{3}^{2-}$ in an environment where pH is increased by Mg (OH)_2_ production [37]. The H^+^ formed in the course of ${\text{HCO}}_{3}^{-}$ → ${\text{CO}}_{3}^{2-}$ as Equation (4) can reduce the pH, so that buffering effect can be obtained and the gas formation can be reduced [38].

Previous studies [39] have reported that H_2_ and CH_4_ occurred from natural metals reacted with water. Therefore, the detected CO after the *in vitro* immersion test seems to be formed by the reaction (CH_4_ + H_2_O ⇔ CO + 3H_2_) between H_2_O and CH_4_,which formed under reducing conditions (2H_2_ + C ⇔ CH_4_) of ${\text{HCO}}_{3}^{-}$ in EBSS [40].

CO is generated as heme oxygenase enzymes are activated in cells once macrophages recognize the bacteria inside the body [41,42]. The dissolved Mg was also absorbed along with CO, which improved the absorption rate of oxygen and reduced the oxidation reaction activity of CO. Thus, dissolved Mg played a role in reducing the concentration of CO [43,44]. It should be noted that a tiny amount of CO can play an important role in cardiovascular systems. It can reduce blood pressure by releasing the smooth muscles located in the blood vessels and inhibit the activity of blood platelet, thereby preventing blood coagulation [45].

Unlike the immersion study*,* the *in vivo* gas formation was rapidly reduced after 10 days. This can be explained via transport of CO_2_ by erythrocytes through the following chemical reaction [36,46];

(5) $${\text{CO}}_{2}+{\;\text{H}}_{2}\text{O}\;\to {\text{HCO}}_{3^{-}}+\;{\text{H}}^{+}$$

The formation of bubble pockets *in vivo* was less intense because the gas generation rate was balanced with the absorption and transport rates of H^+^ and CO_2_ in the body owing to the formation of the natural oxide film. Thus, controlling the initial corrosion rate is a very important factor in Mg tissue bonding and recovery. Generally, the pH is increased locally at the initial degradation interface between body tissue and Mg. Simultaneous increase of pH and Mg ion concentration reaches the critical point and tries to achieve solubility equilibrium of Mg and induces increase of Mg(OH)_2_ as Equation (1). Also in increase in pH near the locally degenerate surface can cause another competitive reaction, precipitation of calcium phosphate compounds such as 5Ca^2+ ^+ $3\text{PO}-4^{3-}$ + OH^−^ ↔ Ca_5_(PO_4_)_3_(OH) [47].

According to the ICP-MS results, the amount of concentration of Mg ions in the liver and kidney slightly increased from 20 to 25 days before reaching the normal value. Mg ions are mainly absorbed in the ileum and colon via passive diffusion, and although some of them are discharged with bile, intestinal fluid and pancreatic juice, most of them are discharged with the urine [48]. Concentration of Mg ions in the kidney decreased up to 15 days and increased thereafter, which means that the accumulation and external discharge of Mg ions follow a certain cycle. The biopsy results revealed that after 15 days, the glomerulus in the globular capillary network showed micro-damage to the renal tubular cells and connective tissue proliferation as a result of renal hematopoietic tissue damage and glomerular contraction [49]. However, the damaged tissues recovered to normal as a result of the circulation and discharge of Mg ions over time. Concentration of Mg ions in the liver initially decreased and increased thereafter as partial vacuolization occurred after 25 days. Local inflammation and mutation were not observed due to the accumulation of metal ions. In previous animal study using Mg alloy pins, it showed to similar results to this study that the implantation into the medullary canal of the femur does not cause measurable side effects on inner organs morphology as liver, spleen and kidney [50]. Another study reported that Mg ions reduce lindane-induced hepatotoxicity and neurotoxicity [51].

Regarding the clinical results, the change of the mean gas volume was similar as compared to *in vivo* studies. The gas volume developed in the first week and then decreased over time. The mean gas volume in the soft tissues was larger than that in bone when the fracture site had a gap. We suggest that this phenomenon could occur by the gas within the bone leaking into the soft tissue through the fracture gap. However, when the fracture heals, there is no gas leakage which would induce an osteolytic lesion around the screw. This occurred in all cases, without delayed unions. An explanation may be that the formation of apatite during Mg degradation may induce bone healing as an osteoconductive effector. In the previous studies, the implantation of Mg metallic glasses into the distal femur increased in cortical-bone thickness and formation of new bone at the peripheral cortex around the periosteum [52,53]. Mg ions were released into the periosteum, which affects magnesium-induced bone formation, by substantially increasing the neuronal calcitonin gene-related polypeptide-α (CGRP) in the peripheral cortex of the femur [54]. This phenomenon can also be applied to magnesium implantation in metatarsal or midfoot fractures. Further study is also required regarding the two cases of wound dehiscence since serious wound complications could occur as the amount of gas released increases with the size of the Mg implant.

## 5. Conclusions

In this study, the gas released during degradation of Mg implants was composed of H_2_, CO and CO_2_. It developed during the initial stages after implantation and was slowly absorbed over time. The Mg ions were not harmful to the kidney or liver, and no serious bone healing complications were observed. However, the gas can induce complications such as superficial skin necrosis and long term osteolytic lesions by clinical trial. Therefore, further studies are required to minimize these complications.

## Disclosure statement

No potential conflict of interest was reported by the authors.

## Funding

This work was supported by a grant of the CNUH-BRI (Biomedical Research Institute of Chonbuk National University Hospital, CNUH-BRI-2012-02-005) and National Research Foundation of Korea (NRF) grant funded by the Korea government (MSIP) [grant numbers 2014R1A4A1005309; 2015R1D1A1A01057144], and this study has reconstructed the data of Seo-Young Kim’s doctoral dissertation.

## Supplemental data

Supplemental data for this article can be accessed here https://doi.org/10.1080/14686996.2018.1451717.

## Supplementary Material

Supplementary_Materials.doc
