# Supplementary material for: Gas formation and biological effects of biodegradable magnesium in a preclinical and clinical observation
Source: Sci Technol Adv Mater. 2018 Apr 9;19(1):324–35. doi: 10.1080/14686996.2018.1451717 (PMC5917435; doi:10.1080/14686996.2018.1451717)
Supplement: Supplementary_Materials.doc [file TSTA_A_1451717_SM4157.doc]

**Supplementary Materials:**


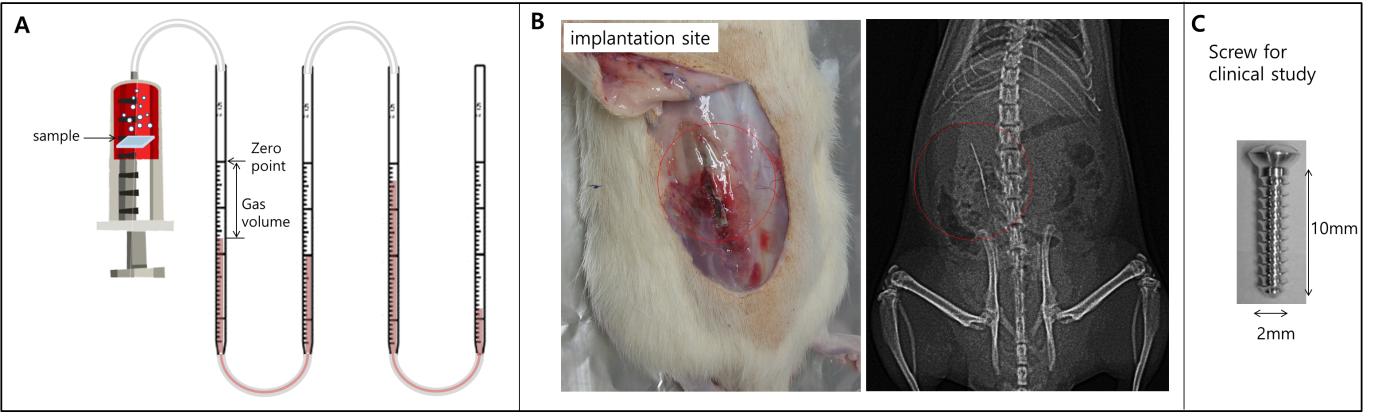


Fig. S1. **A.** Schematic view of the gas formation devices, **B.** Implant surgery model; red circles indicate the implantation site, and **C.** cortex screw for clinical study.


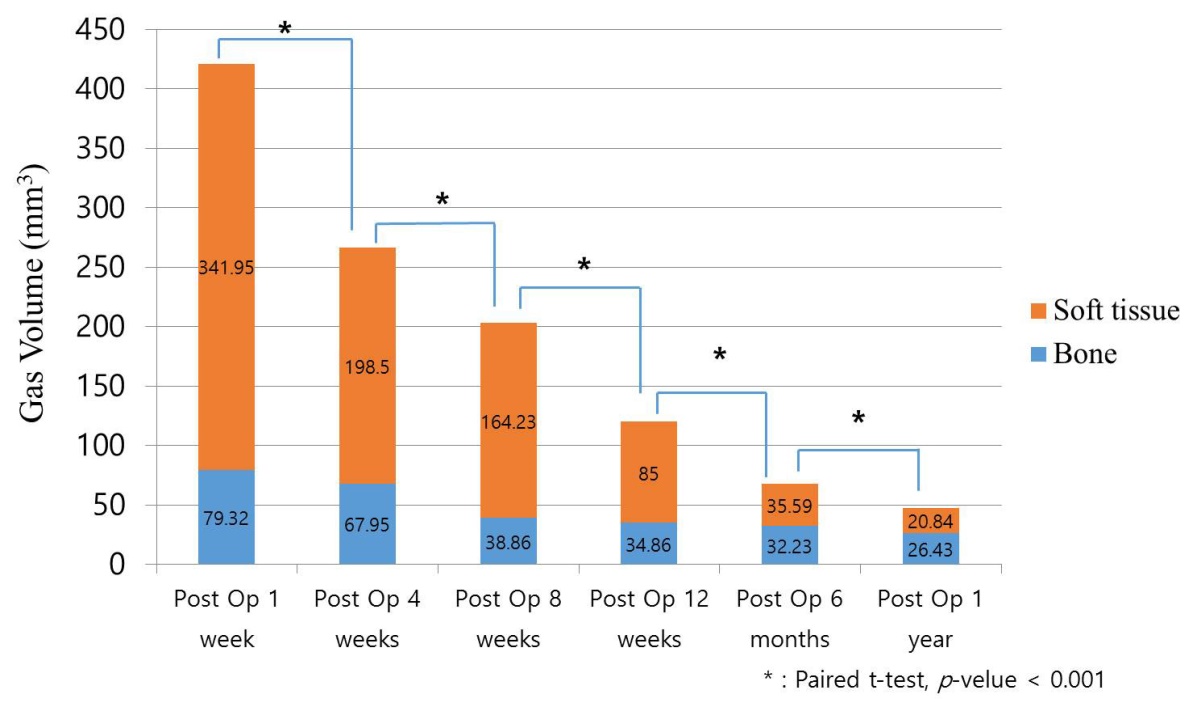


Fig. S2. Total mean gas volume and mean gas volume in both soft tissue and bone showing a significant decrease over time (p < 0.001, p < 0.001 at all each time points, paired t-test).

Table S1. Composition of EBSS (mmol/L)

| Composition | EBSS |
| --- | --- |
| Na^+^  K^+^  Mg^2+^  Ca^2+^  Cl^-^  H_2_PO_4_^-^  SO_4_^2-^  HCO_3_^-^  Glucose  Phenol red | 144  5.4  0.4  1.8  125  1.0  0.4  26  5.6  0.03 |

Table S2. Gas volume in bone, soft tissue and total (mean±S.D, mm^3^) and statistical data in gas volume depending on follow-up time points of clinical trial

| Gas volume in bone, soft tissue and total (mean±S.D, mm^3^) | | | | | | | | | | | |  | | | |  |
| --- | --- | --- | --- | --- | --- | --- | --- | --- | --- | --- | --- | --- | --- | --- | --- | --- |
|  | **Post Op**  **1 week** | **Post Op**  **4 weeks** | | | **Post Op**  **8 weeks** | | | **Post Op**  **12 weeks** | | | **Post Op**  **6 months** | | **Post Op**  **12 months** | | |  |
| **Bone** | 79.32 ± 19.00 | 67.95 ± 15.73 | | | 38.86 ± 7.55 | | | 34.86 ± 6.07 | | | 32.23 ± 5.61 | | 26.43 ± 3.21 | | |  |
| **Soft tissue** | 341.95 ± 126.42 | 198.50 ± 84.47 | | | 164.23 ± 70.21 | | | 85.00 ± 42.95 | | | 35.59 ± 37.79 | | 20.84 ± 31.17 | | |  |
| **Total** | 421.27 ± 143.47 | 266.45 ± 97.98 | | | 203.09 ± 75.37 | | | 119.86 ± 46.04 | | | 77.82 ± 39.36 | | 47.27 ± 34.38 | | |  |
|  | | | | | | |  | | | | | | | | |  |
| Statistical data in gas volume depending on follow-up time points | | | | | | | | | | | | | | |  |  |
| **Comparison** | | | **p value** | **p value** | | | | | **Comparison** | | | **p value** | | |  |  |
|  | | | **Bone** | **Soft tissue** | | | | | **Bone vs. Soft tissue** | | |  | | |  |  |
| 1 week vs. 4 weeks | | | <0.001 | | | <0.001 | | | | 1 week vs. 1 week | | | | <0.001 | | |
| 4 weeks vs. 8 weeks | | | <0.001 | | | 0.006 | | | | 4 weeks vs. 4 weeks | | | | <0.001 | | |
| 8 weeks vs. 12 weeks | | | <0.001 | | | <0.001 | | | | 8 weeks vs. 8 weeks | | | | <0.001 | | |
| 12 weeks vs. 6 months | | | <0.001 | | | <0.001 | | | | 12 weeks vs. 12 weeks | | | | <0.001 | | |
| 6 months vs. 12 months | | | <0.001 | <0.001 | | | | | | 6 months vs. 6 months | | | | 0.105 | | |
|  | | |  |  | | | | | | 12 months vs. 12months | | | | <0.001 | | |

Table S3. Analysis of the American Orthopaedic Foot and Ankle Society (AOFAS) Ankle-Hindfoot Score, and the visual analog scale (VAS)

|  | Pre Op | Post Op 1 week | Post Op 4 week | Post Op 8 weeks | Post Op 6 months | Post Op 1 year | *p*-value |
| --- | --- | --- | --- | --- | --- | --- | --- |
| VAS | 7.14 ± 0.77 | 2.77 ± 0.75 | 2.32 ± 0.63 | 2.02 ± 0.82 | 1.84 ± 0.43 | 1.77 ± 0.75 | < 0.0001 |
| AOFAS score | 41.72 ± 7.54 | 76.92 ± 6.87 | 80.21 ± 4.23 | 84.51 ± 4.82 | 86.36 ±3.62 | 88.91 ± 3.52 | < 0.0001 |
